# The Identification of Two RNA Modification Patterns and Tumor Microenvironment Infiltration Characterization of Lung Adenocarcinoma

**DOI:** 10.3389/fgene.2022.761681

**Published:** 2022-01-28

**Authors:** Wan He, Gengpeng Lin, Chaohu Pan, Wenwen Li, Jing Shen, Yangli Liu, Hui Li, Dongfang Wu, Xuejia Lin

**Affiliations:** ^1^ Department of Oncology, Shenzhen People’s Hospital, Shenzhen, China; ^2^ Department of Pulmonary and Critical Care Medicine, The First Affiliated Hospital of Sun Yat-sen University, Institute of Pulmonary Diseases, Sun Yat-sen University, Guangzhou, China; ^3^ Zhuhai Institute of Translational Medicine, Zhuhai People’s Hospital (Zhuhai Hospital Affiliated With Jinan University), Jinan University, Zhuhai, China; ^4^ The Biomedical Translational Research Institute, Faculty of Medical Science, Jinan University, Guangzhou, China; ^5^ YuceBio Technology Co., Ltd., Shenzhen, China; ^6^ Department of Pathology, The First Affiliated Hospital of Sun Yat-sen University, Guangzhou, China

**Keywords:** RNA modification “writers”, lung adenocarcinoma, WM score, tumor microenvironment, immunotherapy

## Abstract

**Background:** RNA modification plays an important role in many diseases. A comprehensive study of tumor microenvironment (TME) characteristics mediated by RNA modification regulators will improve the understanding of TME immune regulation.

**Methods:** We selected 26 RNA modification “writers” of lung adenocarcinoma (LUAD) samples and performed unsupervised clustering analysis to explore RNA modification patterns in LUAD. Differentially expressed genes (DEGs) with RNA modification patterns were screened to develop a “writers” of RNA modification score (WM score) system. The infiltration ratio of TME cell subsets was analyzed by CIBERSORT.

**Results:** We identified two RNA modification modes showing different characteristics of overall survival (OS) and TME cell infiltration. According to WM score, LUAD patients were divided into a high-WM score group and a low-WM score group. High-scored patients had a poor prognosis and higher tumor mutation burden (TMB), they were more sensitive to four LUAD therapies (erlotinib, XA V939, gefitinib, and KU-55933) and more clinically responsive to PD-L1 treatment. Those with a low WM score showed higher stromal scores, ESTIMATE scores, and survival chance.

**Conclusion:** Our work revealed the potential role of RNA modification patterns in TME, genetic variation, targeted inhibitor therapy, and immunotherapy. Identifying RNA modification pattern of LUAD patients help understand the characteristics of TME and may promote the development of immunotherapy strategies.

## Introduction

The mortality of lung cancer far exceeds that resulting from breast cancer, pancreatic cancer, colon cancer, and prostate cancer (Siegel et al., 2018). Lung adenocarcinoma (LUAD) as a subtype of lung cancer evolves from mucous glands, and is found in many areas with scars or chronic inflammation (Myers and Wallen, 2021). Chronic dry cough, dyspnea, hemoptysis, and weight loss are main manifestations of LUAD (Mullangi and Lekkala, 2021). LUAD accounts for almost half of all lung cancer deaths, with a 5-year survival rate as low as 15% (Kara et al., 2021; Spella and Stathopoulos, 2021). A large proportion of LUAD patients have already developed metastasis by the time of diagnosis (Siegel et al., 2021), but available treatments for those patients are limited and often challenging.

Cancer development may be driven by genetic and epigenetic aberrations and complex crosstalk between different pathways (Spella and Stathopoulos, 2021). RNA-modification and enzymes that catalyze RNA modification (including writers, erasers, and readers) contribute to precursor mRNA splicing, nuclear output, transcriptional stability, and translation initiation of eukaryotic cells (Shi et al., 2020). Several types of RNA modifications affecting the processing and function of different RNA types have been reported, for example, methylation (N^7^-methylguanosine [m^7^G], N6-methyl-2′-O-methyladenosine [m^6^Am], 2′-O-m ethylation [Nm], N6-methyladenosine [m^6^A], N1-methyladenosine [m^1^A], 5-methylcytosine [m^5^C] and 5-hydroxymethylcytosine [hm^5^C]), and RNA editing [adenosine-to-inosine (A-to-I), pseudo-uridine(Ψ)] (Lobo et al., 2018). Among a wide range of RNA modifications, on adenine, including m^6^A, m1A, alternative polyadenylation (APA) (Soles and Shi, 2021), and A-to-I, is the most common, and they are mainly regulated by RNA modified “writers” (Muthusamy, 2020). Among them, M^6^A modification, which is the most common modification in transcripts in the common sequence RRm^6^ACH (Zhao et al., 2017), refers to the methylation of adenosine base at the nitrogen-6 position. M^1^A adds a methyl and a positive charge to adenosine N1 to block the Watson-Crick interface, which will change the secondary structure of RNA and protein-RNA interaction (Xie et al., 2020). APA is an mRNA-related process that produces multiple transcriptional subtypes through selecting alternate (proximal or distal) polyadenylation signals on the 3′-UTR of pre-mRNAs and even proteomic diversity (Akman and Erson-Bensan, 2014). A-to-I editing consists of the irreversible conversion of adenosine to inosine catalyzed by adenosine deaminase acting on RNA (ADAR) enzymes (Marceca et al., 2021). These RNA modification patterns participate in various physiological processes and play important regulatory roles in diseases including cancers, neurologic and metabolic diseases (Wilkinson et al., 2021).

This study examines the patterns of RNA modification integrating clinicopathological information and genomic data from 739 LUAD samples. The relationship between RNA modification patterns, genetic mutation, and the characteristics of TME cell infiltration was also analyzed. Moreover, we developed a scoring system to quantify the RNA modification of individual LUAD samples for predicting clinical responses of LUAD patients to chemotherapy and immunotherapy.

## Materials and Methods

### Data Acquisition and Collation

The expression data and clinical data of LUAD patients in the GSE31210 cohort and GSE72094 cohort were obtained from the GEO database. Expression data (mRNA expression, miRNA expression), genomic mutation data (somatic mutation, somatic copy number change (SCNA), and clinical information (tumor stage, histological subtype, sex, and total survival time) of 513 patients with LUAD were downloaded from TCGA database (https://portal.gdc.cancer.gov/) on 18 May 2021.

### Consensus Clustering for 26 RNA Modification “Writers”

A total of 26 RNA modification “writers”, including 7 m^6^A modification enzymes (METTL3, METTL14, WTAP, RBM15, RBM15B, ZC3H13, and KIAA1429), 4 m^1^A modification enzymes (TRMT61A, TRMT61B, TRMT10C, and TRMT6), 12 APA modification enzymes (CPSF1-4, CSTF1/2/3, PCF11, CFI, CLP1, NUDT21, and PABPN1) and 3 A-I modification enzymes (ADAR, ADARB1, and ADARB2), were obtained from previously published studies. ConsensusClusterPlus (maxK = 10, reps = 10, pItem = 0.8, pFeature = 1, clusterAlg = “hc” innerLinkage = “average”, finalLinkage = “average”, distance = “pearson”) (Wilkerson and Hayes, 2010) was employed in unsupervised clustering analysis on the RNA modification “writers” of the samples in GSE31210 cohort.

### Gene Set Variation Analysis and Functional Annotation

GSVA enrichment analysis was performed in R package “GSVA” (method = ssgsea, kcdf = Gaussian) (Hanzelmann et al., 2013) to analyze the biological processes in which different RNA modification “writers” were enriched. Adjusted P by Benjamini and Hochberg with a value less than 0.001 was considered to be statistically significant. The hallmark gene set was downloaded from the MSigDB database (Liberzon et al., 2015). Functional annotation of 26 RNA modification “writers” was performed using clusterProfiler (minGSSize = 10, maxGSSize = 500, qvalueCutoff = 0.2) (Yu et al., 2012), false discovery rate (FDR) was adjusted by Benjamini and Hochberg, and the cutoff value of FDR <0.05 was set.

### Immune Cell Abundance Estimation by CIBERSORT

CIBERSORT (Newman et al., 2015) was employed to predict the immune score of 22 kinds of immune cells in a LUAD microenvironment with support vector regression (SVR). In here, deconvo_cibersort function of R software package “IOBR” (perm = 1,000, abs_method = “sig.score”) was conducted for above analysis. This is a machine learning approach that improves deconvolution performance through a combination of feature selection and robust mathematical optimization techniques (Chen et al., 2018).

### Construction of the “Writer” of RNA Modification Score

To develop a WM scoring system, differentially expressed genes (DEGs) among different RNA modification patterns using a linear model with the limma package (the significance criteria for determining DEGs was set to FDR <0.01 and log fold-change >1.0), and those related to LUAD survival were identified by univariate COX regression analysis. Functional enrichment analysis of survival-related DEGs was carried out using the clusterProfiler software package (the cutoff value of FDR <0.05). The coefficients of each gene were determined by univariate Cox regression analysis, and we developed a formula for calculating WM score similar to a previous study (Sotiriou et al., 2006) as follows:

WM score= βi×Xi.

β*i* was the coefficients of each gene determined by univariate Cox regression analysis, and X*i* was the expression level of the RNA modification phenotype-related genes.

### Analysis of Post-Transcriptional Regulation of WM Score

The WM score of each LUAD sample was calculated and the critical score was determined according to surv_cutpoint function of R software package “survminer” (minprop = 0.1). The LUAD patients were divided into high-WM score group and low-WM score group. Differential miRNAs between the two groups were obtained by differential miRNA analysis, and their targets were predicted by TargetScan (http://www.targetscan.org/vert_72/). KEGG was used to analyze the signal pathway in which the differential miRNA target genes were enriched. The calculation was conducted with the clusterProfiler package, and statistical significance was set at 0.05.

### Correlation Analysis Between WM Score and Chemotherapeutic or Immune Checkpoint Blocking Therapy

Drug sensitivity data of about 1,000 cancer cell lines were downloaded from Genomics of Drug Sensitivity in Cancer (GDSC, http://www.cancerrxgene.org). The R package pRRophetic (drug = erlotinib, tissueType = urogenital_system, selection = 1, dataset = cgp 2016) was used to examine chemotherapeutic response determined as the half-maximal inhibitory concentration (IC_50_) of each LUAD sample in the GDSC website. The correlation between drug sensitivity and WM score was analyzed by Spearman. Two immunotherapy cohorts [IMvigor210 (Mariathasan et al., 2018) and GSE78220 cohort (Hugo et al., 2016)] were also included. The relationship between WM score and patients’ response to immune checkpoint blocking therapy was analyzed.

### Statistical Analysis

All the statistical analyses of this study were conducted in R program (version R3.6.2). One-way ANOVA and Kruskal–Wallis tests were applied for comparing differences. The receiver operating characteristic (ROC) curve was used to determine the specificity and sensitivity of the WM score. In addition, independent prognostic factors were screened by multivariate Cox regression analysis. FDR was adjusted by Benjamini and Hochberg to reduce te false-positive rate in multiple tests. All statistical *p* values were two-sided. *p* < 0.05 indicated statistical significance.

## Results

### TME Characterization of LUAD and Genetic and Expression Changes of RNA Modification “Writers”

**FIGURE 1:**
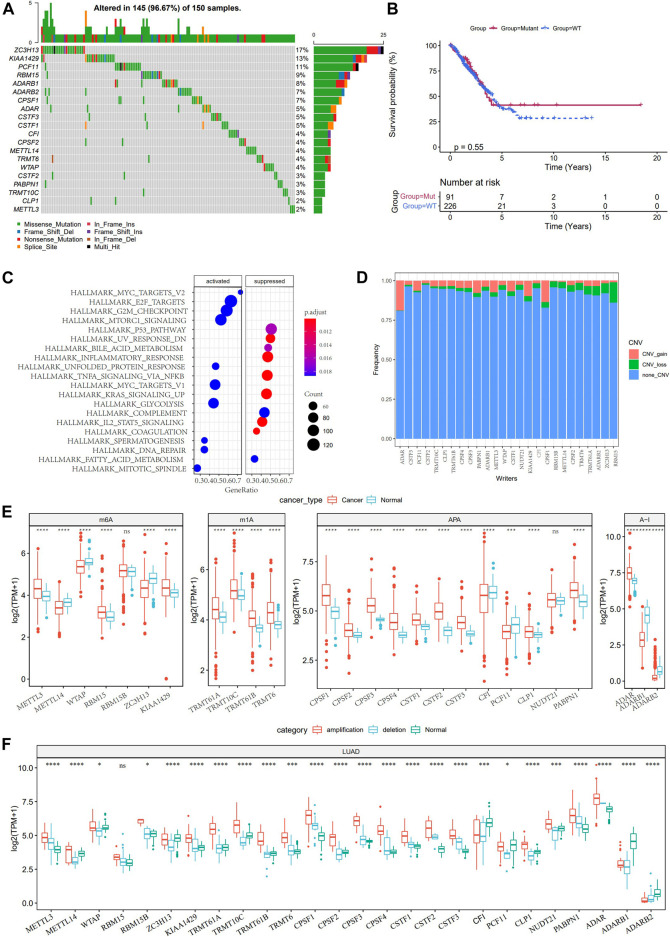
Genetic and expression changes of RNA modification “writers” in LUAD. **(A)**: 20 RNA modification “writers” with the highest mutation frequency. **(B)**: The difference of overall survival between LUAD patients with RNA modification “writers” mutation and those without mutation. **(C)**: GSVA analysis showed the enrichment pathway of mutant RNA modification “writers”. **(D)**: The frequency of CNV gain, loss, and none CNV of RNA modification “writers” in the TCGA-LUAD database. **(E)**: Analysis of the difference of 26 RNA modification “writers” between normal and LUAD tissues. **(F)**: The expression of RNA modification “writers” in LUAD patients was divided into groups based on CNV value. *p < 0.05; **p < 0.01; ***p < 0.001; ****p < 0.0001.

Considering the dual role of TME in regulating LUAD development, we analyzed the TME characteristics between normal tissues and LUAD tissues in TCGA. According to the results of the ESTIMATE algorithm, the immune score, stromal score, and ESTIMATE score of LUAD were significantly lower than those of normal tissues (Supplementary Figure S1). Then, we studied the somatic mutation and copy number variations (CNV) of 26 RNA modification “writers”. Mutations of RNA modification “writers” were detected in 42 TCGA-LUAD samples. Among all the mutant RNA modification “writers”, those with the highest mutation frequency were ZC3H13 (17%), KIAA1429 (13%), and PCF11 (11%), respectively (Figure 1A). No significant statistical difference was found in the overall survival (OS) of LUAD patients with or without these RNA modification “writer” mutations, suggesting that “writer” mutations may have a limited effect on the overall survival of LUAD patients (Figure 1B). GSVA enrichment analysis showed that the mutant “writers” were mainly enriched in carcinogenic pathways such as MYC targets, E2F targets, mTORC1 signaling, and G2M checkpoint, indicating that the mutation of “writers” could affect the regulation of multiple signals (Figure 1C). CNV analysis showed that the amplification in copy number of ADAR and CPSF1 was the most extensive, while RBM15B, ZC3H13, ADARB2, and TRMT61A showed great CNV deletion, and no CNV occurred in KIAA1429 (Figure 1D). Further study on the expression of RNA modification “writers” between normal tissues and LUAD tissues demonstrated that apart from RBM15B and NUDT21, there were significant differences in the expression of 24 RNA modification “writers” (Figure 1E). According to the CNV value, the LUAD patients were divided into CNV amplification group, CNV deletion group, and normal group. Figure 1F showed that the expression of RNA modification “writers” in the CNV amplification group was higher than that in the CNV deletion group.

### Identification of Two Patterns of RNA Modification “Writers” and Analysis of the Characteristics of TME Cell Infiltration

**FIGURE 2:**
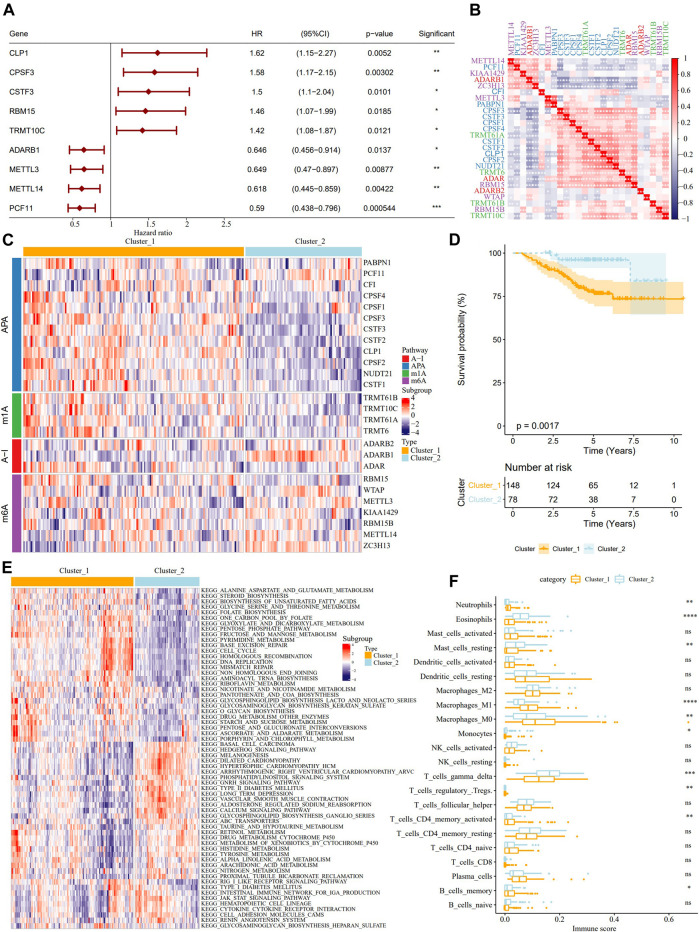
Identification of the characteristics of TME cell infiltration by two patterns of RNA modification “writers” and analysis. **(A)**: The forest map showed 9 RNA modification “writers” significant in the prognosis of LUAD. **(B)**: Correlation analysis among RNA modification “writers”. **(C)**: According to the expression of RNA modification “writers”, the samples in GSE31210 data sets were clustered. **(D)**: The difference of OS between the two kinds of RNA modification patterns. **(E)**: Biological pathway of GSVA enrichment analysis to determine the enrichment of cluster_1 and cluster_2. **(F)**: The immune score difference of TME cells between the two kinds of RNA modification patterns. *p < 0.05; **p < 0.01; ***p < 0.001; ****p < 0.0001.

Univariate Cox analysis on the samples from the GSE31210 dataset identified 9 RNA modification “writers” with prognostic significance in LUAD (Figure 2A). The results of pairwise correlation analysis showed a significant correlation between most RNA modification “writers” (Figure 2B), and their internal connections may have critical functions in the RNA-modified tumor model. According to the expression of RNA modification “writers”, ConsensusClusterPlus was used to classify the samples in the GSE31210 dataset, and two RNA modification modes, cluster_1 (148 LUAD patients) and cluster_2 (78 LUAD patients), were determined (Figure 2C). From the survival analysis of two RNA modification subtypes, we found that samples with the cluster_2 RNA modification pattern showed better survival results (Figure 2D). The biological pathways of the two clusters were examined by GSVA enrichment analysis, and the data revealed that cluster_1 was significantly enriched in cell cycle, cell division, and metabolic pathways, while cluster_2 was more associated with diseases such as heart disease and diabetes (Figure 2E). Then we further analyzed the correlation between RNA modification “writers” and TME cells, and each RNA modification “writer” was found to be related to different immune cells (Supplementary Figure S2). Additionally, differences in neutrophils, eosinophils, resting mast cells, M0 macrophages, M1 macrophages, monocytes, gamma delta T cells, regulatory T cells, activated memory CD4 T cells, memory B cells were also found between the two RNA modification patterns. The immune scores of neutrophils, M0 macrophages, M1 macrophages, activated memory CD4 T cells, and memory B cells were significantly higher in Cluster_1 than in Cluster_2. However, the immune score of eosinophils, resting mast cells, monocytes, gamma delta T cells, and activated memory CD4 T cells was significantly lower than in cluster_2 (Figure 2F).

### Development and Validation of RNA Modification “Writers” Scores

**FIGURE 3:**
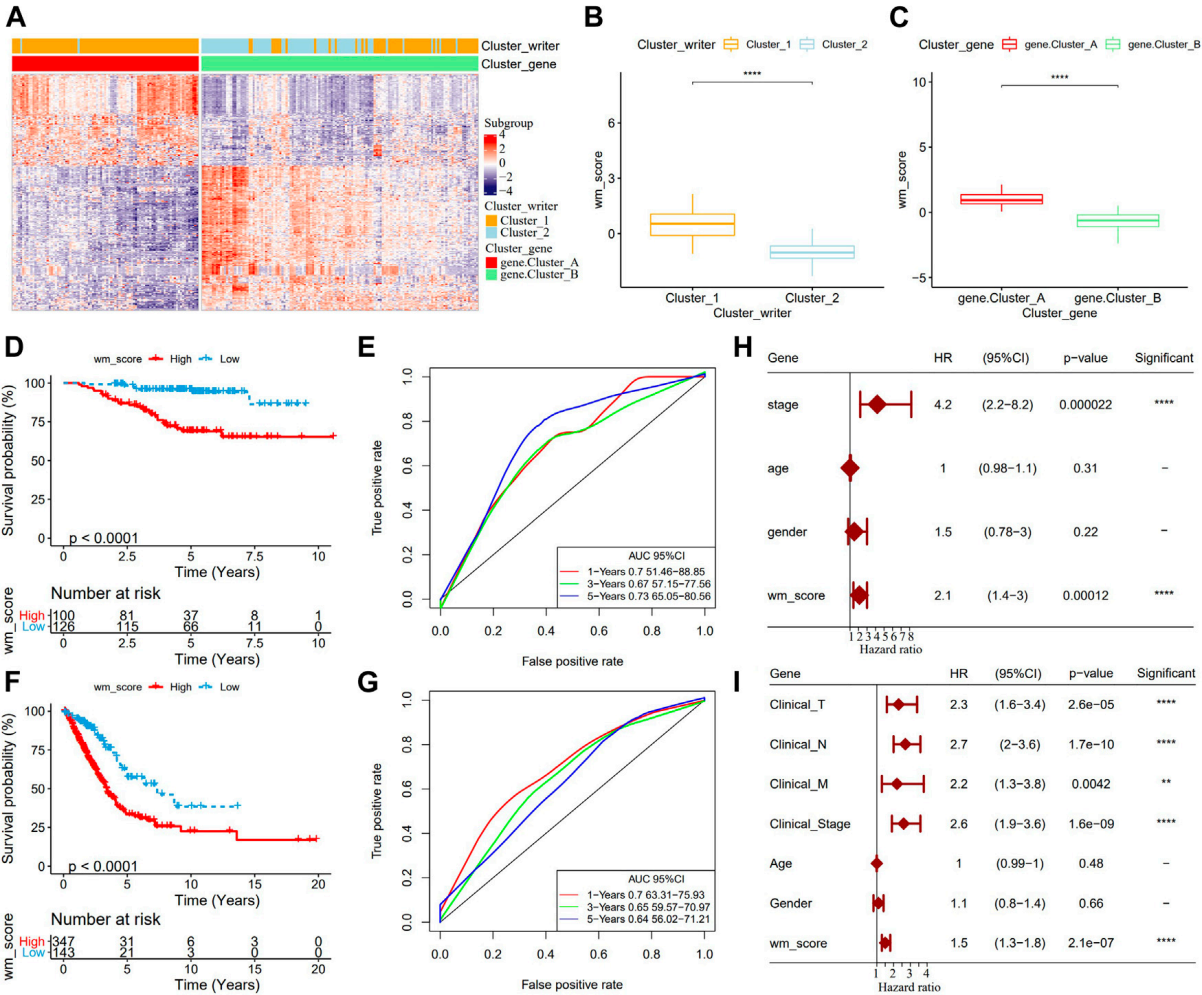
Development and validation of RNA modification “writers” scores. **(A)**: The heat map of DEGs. **(B)**: WM score differences between different RNA modification pattern clusters. **(C)**: WM score differences between the two genomic subtypes. **(D)**: In the GSE31210 cohort, OS differences between patients with high WM scores and patients with low WM scores. **(E)**: Time-dependent ROC curve of WM score in GSE31210 cohort. **(F)**: In the GSE31210 cohort, Kaplan-Meier curves of patients with high WM score and low WM score. **(G)**: Time-dependent ROC curve of WM Score in TCGA-LUAD cohort. **(H)**: Univariate Cox analysis to determine the relationship between clinical variables and OS in patients with LUAD in the GSE31210 cohort. **(I)**: Univariate Cox analysis predicted the correlation between clinicopathological factors and prognosis of patients with LUAD in TCGA-LUAD data set. **p < 0.01; ****p < 0.0001.

Although our study analyzed the role of RNA modification patterns in tumor development and immune infiltration regulation, the results were based on patient group studies and may not be able to accurately predict the pattern of RNA modification “writers” in a single LUAD sample. Therefore, we developed WM score, a scoring scheme for determining the RNA modification pattern of a single LUAD sample. Firstly, 269 differentially expressed genes (DEGs) related to RNA phenotype were obtained through differential analysis. According to the results of unsupervised cluster analysis on the 269 DEGs, consistent with the cluster grouping of RNA modification pattern, LUAD was divided into two genomic subtypes (gene.cluster A and gene.cluster B) (Figure 3A). From the two box diagrams of WM score between different RNA modification pattern cluster and genomic subtypes, it could be observed that the WM score of cluster_1 was significantly higher than that of cluster_2, and similarly the WM score of gene.cluster A was also significantly higher than that of gene.cluster B (Figures 3B,C). More importantly, in the GSE31210 cohort, the prognosis of patients with high WM scores was worse than those with low scores (Figure 3D). The AUC of time-dependent ROC curve of WM score in 1 year, 3 years, and 5 years were all greater than 0.65 (Figure 3E). To further verify the reliability of the WM score model, we used TCGA-LUAD queues to determine the relationship between WM score and patients’ OS. Consistent with the results of the GSE31210 cohort analysis, the survival of patients in the TCGA-LUAD cohort with high WM scores was significantly lower than those with low WM scores (Figure 3F). The 1-year, 3-year, and 5-year AUC of the time-dependent ROC curve of WM score were 0.7, 0.65, and 0.64, respectively (Figure 3G). We also introduced the WM score model into the GSE72094 cohort to calculate the WM score of each sample. In this cohort, the mortality rate of samples with high WM scores increased significantly (Supplementary Figure S3A), and the AUC of the 5-year OS was predicted to be as high as 0.81 (Supplementary Figure S3B). Univariate Cox regression analysis on the GSE31210 and the TCGA-LUAD cohorts demonstrated that WM score could independently predict the prognosis of LUAD (Figure 3F). These results indicated that the WM score can reflect the RNA modification pattern of LUAD patients and effectively predict the prognosis of LUAD.

### The Relationship Between WM Score and TME was Characterized

**FIGURE 4:**
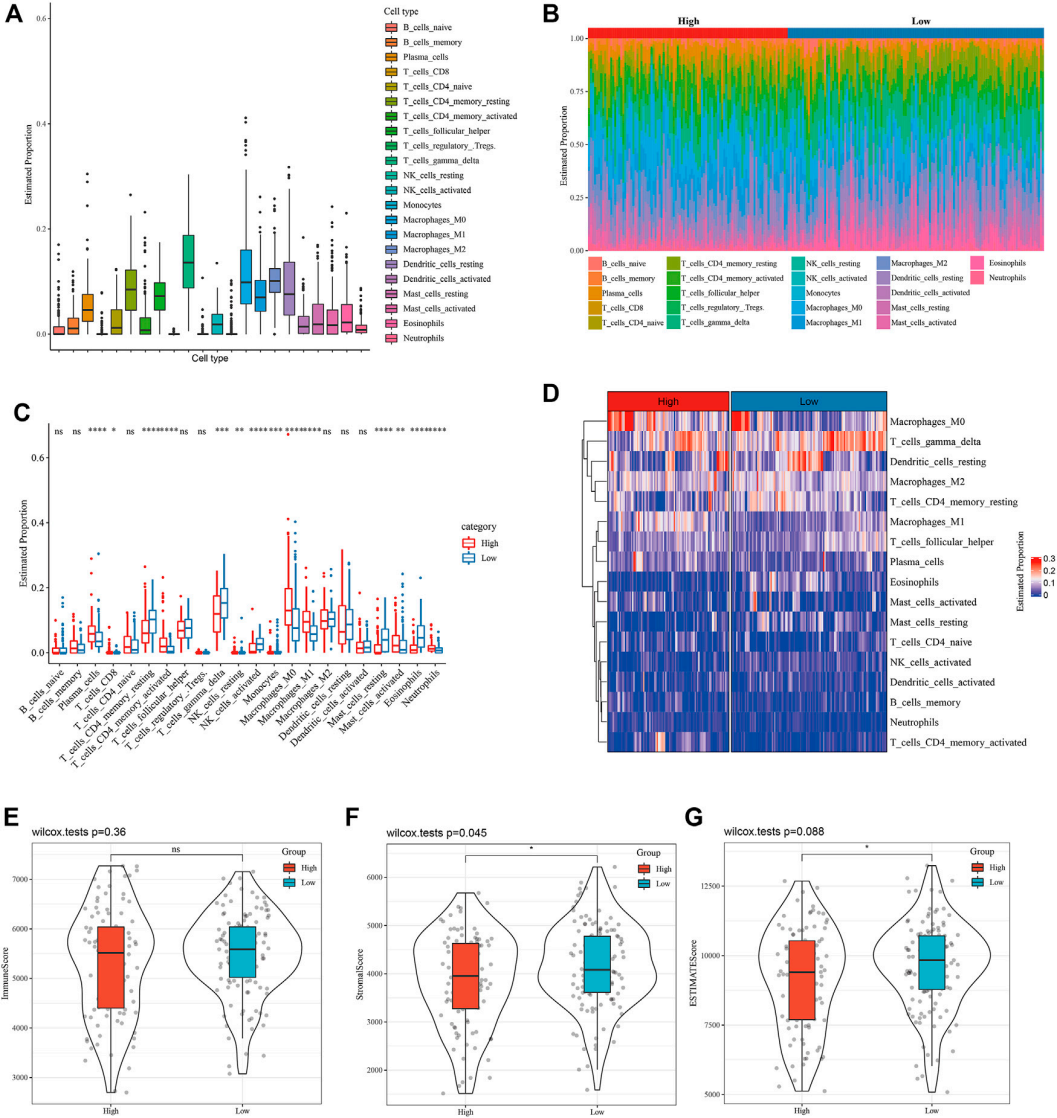
The relationship between WM score and TME was characterized. **(A)**: Proportion of 22 kinds of tumor-infiltrating immune cells (TIIC) in LUAD tissue. **(B)**: The distribution of 22 kinds of TIIC in tumor tissues with high WM scores and low WM scores. Each bar chart represents the relative proportion of TIIC in an organization. **(C)**: Box diagram of TIIC ratio between high WM score and low WM score tumors. Red represents high WM score LUAD organization and red represents low WM score LUAD organization. **(D)**: Heatmap of TIIC proportion in tumor tissues with high WM score and those with low WM score. **(E)**: Immune score between patients with two types of WM score. **(F)**: Stromal score between LUAD patients with high and low WM score. **(G)**: The difference of ESTIMATE score between high and low WM score tumor tissues. *p < 0.05; **p < 0.01; ***p < 0.001; ****p < 0.0001.

The proportion of 22 kinds of tumor-infiltrating immune cells (TIIC) in LUAD tissue was evaluated to help characterize the relationship between WM score and TME, and we found that the proportion of gamma delta T cells was the highest among 22 kinds of TIICs (Figure 4A). The infiltration of immune cells with different WM scores in the GSE31210 data set was studied. From Figure 4B and Figure 4C, it could be observed that there was a significant difference in the proportion of 14 kinds of TIICs (plasma cells, CD8 T cells, resting CD4 memory T cells, activated memory CD4 T cells, gamma delta T cells, resting NK cells, activated NK cells, monocytes, M0 macrophage, M1 macrophage, resting mast cells, activated mast cells, eosinophils and neutrophils) between patients with high- and low-WM score. From the heatmap of TIIC ratio, the proportion of most TIIC between high-WM score and low-TIIC was clearly different (Figure 4D). There was no statistical difference in immune scores between patients with two WM scores (Figure 4E). Significant differences between high and low WM scores were identified, and stromal score and ESTIMATE score were higher in patients with low WM scores (Figures 4F,G). These results indicated that high WM scores and low WM scores showed different TME characteristics.

### Clinical, Somatic Mutation, and Post-Transcriptional Modification Characteristics of WM Score

**FIGURE 5:**
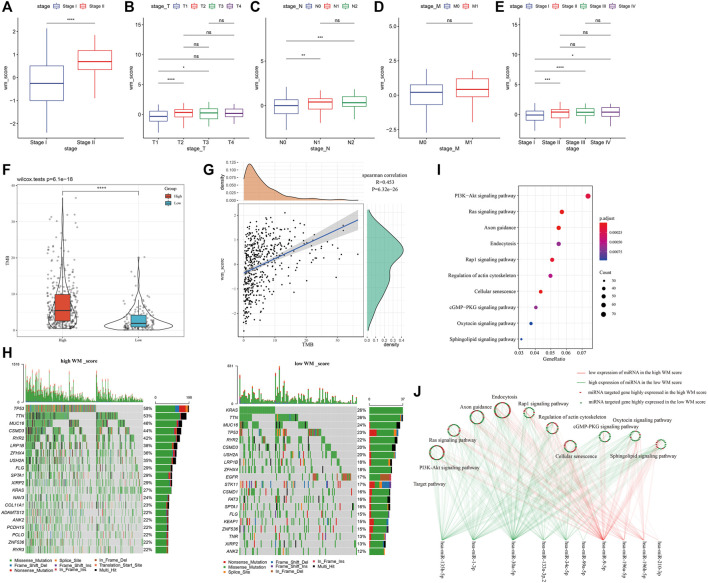
Clinical, somatic mutation and post-transcriptional modification of WM score. **(A)**: In the GSE31210 cohort, the WM score of stage II was significantly higher than that of stage I. **(B)**: In TCGA-LUAD data sets, the WM score of stage II, stage III, or stage IV was higher than that of stage I. **(C)**: In the TCGA-LUAD cohort, WM score analysis according to T stage group. **(D)**: In the TCGA-LUAD cohort, WM score analysis according to N stage group. **(E)**: The difference in WM score between patients with M0 and M1 stage in the TCGA-LUAD data set. **(F)**: Violin pictures of TMB in high WM score and low WM score groups. **(G)**: The correlation between TMB and WM score. **(H)**: Mutated gene analysis between high WM score and low WM score LUAD samples. **(I)**: KEGG enrichment analysis of target genes of miRNA. **(J)**: The relationship between miRNA-mRNA and 10 pathways between high WM score group and low WM score group. *p < 0.05; **p < 0.01; ***p < 0.001; ****p < 0.0001.

To study WM score in different clinical characteristics, LUAD were grouped according to the clinical variables of the GSE31210 and TCGA-LUAD datasets to analyze the WM score differences in different LUAD subgroups. In AJCC stage grouping, the WM score of stage II was significantly higher than that of stage I (Figure 5A). Similarly, in TCGA-LUAD datasets, the WM score of stage II, stage III, or stage IV was higher than that of stage I (Figure 5B). According to the WM score analysis of T stage grouping, the WM score of T2 or T3 was significantly better than that of T1 (Figure 5C). Patients with the N1 or N2 stage showed a noticeably higher WM score than those with the N0 stage (Figure 5D). For M0 and M1, there was no significant difference in WM score between the two (Figure 5E).

The difference in TMB between high-WM score and low-WM score patients in the TCGA-LUAD cohort was also investigated. From the violin map in Figure 5F, it could be observed that patients with high WM scores had higher TMB, and there was a significant positive correlation between TMB and WM score (Figure 5G). Furthermore, mutated gene analysis was carried out on LUAD samples with high- and low-WM scores. The results showed that TP53 (58%), TTN (53%), and MUC16 (46%) had higher somatic mutation rates in the high-WM score group. In low-WM score group, the top genes with the highest mutation frequency were KRAS (26%), TTN (26%), and MUC16 (24%), showing that the high-WM score group had more tumor mutation burden than the low-WM score group (Figure 5H).

Normally, RNA modification “writers” selectively install the code of the entire transcriptional group and set it as the upstream of information processing (Wang and He, 2014). Transcriptional modifications regulated by RNA modification “writers” affect almost every step of RNA metabolism, including mRNA processing, mRNA transfer from nucleus to cytoplasm, mRNA translation, mRNA decay, and biogenesis of microRNAs (miRNAs) (Dai et al., 2018). Analysis of the differences of miRNAs between high- and low-WM scores detected 25 differential miRNAs (Supplementary Table S1). The relationship between miRNA-mRNA and the above 10 pathways between the two WM score groups was shown. The difference between the high-WM score and low-WM score target genes of miRNAs was shown in Figure 5J. The results here suggested that the WM score was related to molecular mutation, expression of miRNAs, and the regulation of signal pathways.

### WM Score Could Predict the Response of Cell Line Drug Therapy and Immunotherapy

**FIGURE 6:**
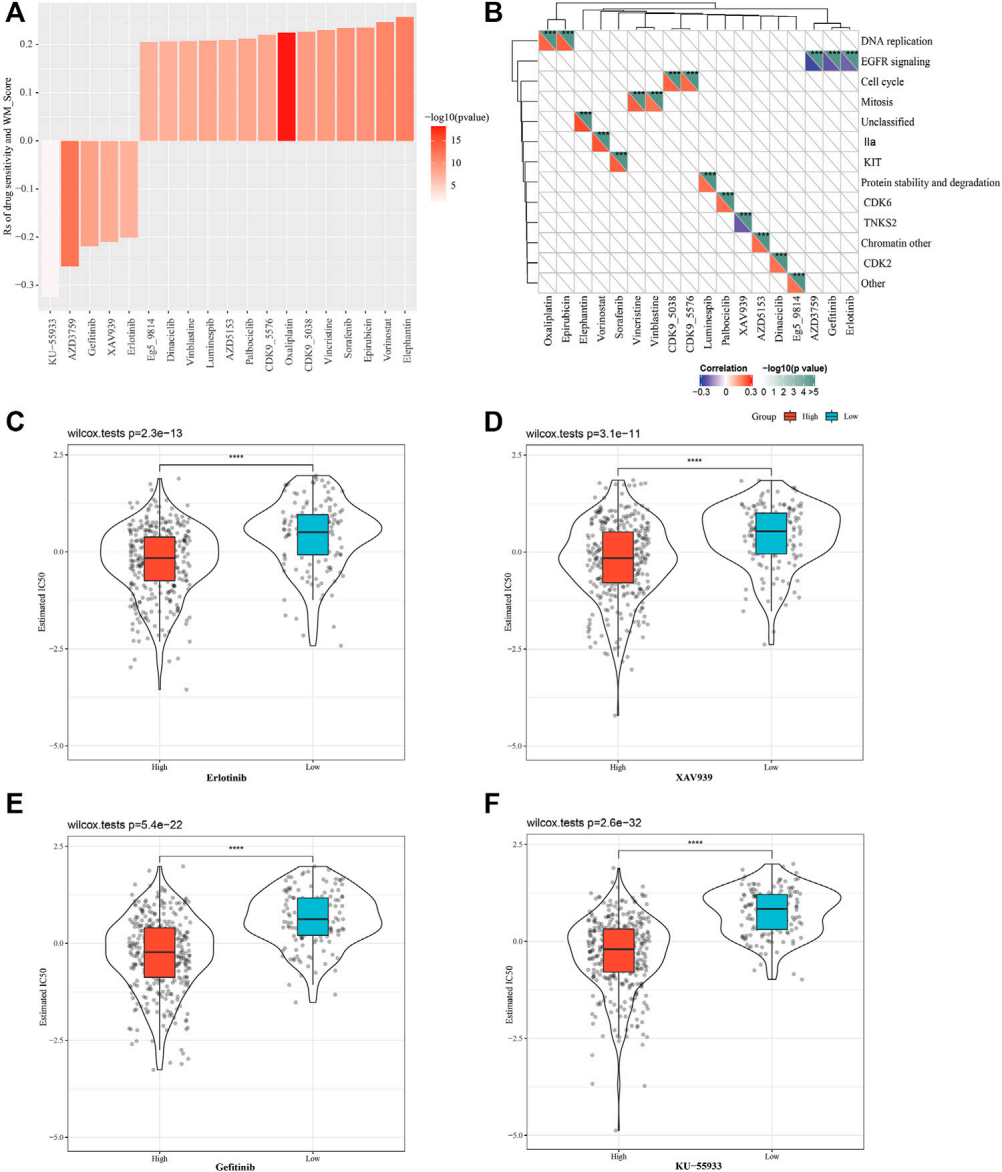
Evaluating the therapeutic response of the different WM score. **(A)**: The relationship between WM score and tumor cell line drug response. **(B)**: Signal pathways regulated by drugs that are resistant or sensitive to WM score. **(C)**: Erlotinib response based on IC50 available in the GDSC database in different WM scores. **(D)**: The IC50 of XAV939 was compared between the high WM score and low-WM score group. **(E)**: The IC50 of gefitinib was compared between the high WM score and low-WM score group. **(F)**: KU-55933 sensitivity based on IC50 available in the GDSC database in different WM scores. ***p < 0.001; ****p < 0.0001.

We also predicted the response of the two WM score groups to conventional drug therapy. Based on Spearman correlation analysis, a total of 19 of the responses to drugs were found to be significantly linked with WM scores in GDSC, specifically, there were 5 drug sensitivities related to the WM score, and resistance to 14 drugs was associated with the WM score (Figure 6A). Analysis of the signaling pathways of the genes regulated by these drugs demonstrated that drug sensitivity associated with the WM score mainly regulated EGFR signaling pathway, and that drug resistance related to the WM score mainly targeted the regulation of DNA replication, cell cycle, mitosis, and other processes (Figure 6B). The drug response was then evaluated based on the half-maximum inhibitory concentration (IC_50_) of each TCGA-LUAD sample in the GDSC database. The estimated IC_50_ of the four tumor inhibitors erlotinib, XAV939, gefitinib, and KU-55933 showed significant differences between the high-WM score group and the low-WM score group, and a lower IC_50_ value was found in the high-WM score group, suggesting that the samples with a high WM score were more sensitive to chemotherapy (Figures 6C–F). The results validated that WM score was associated with drug sensitivity.

**FIGURE 7:**
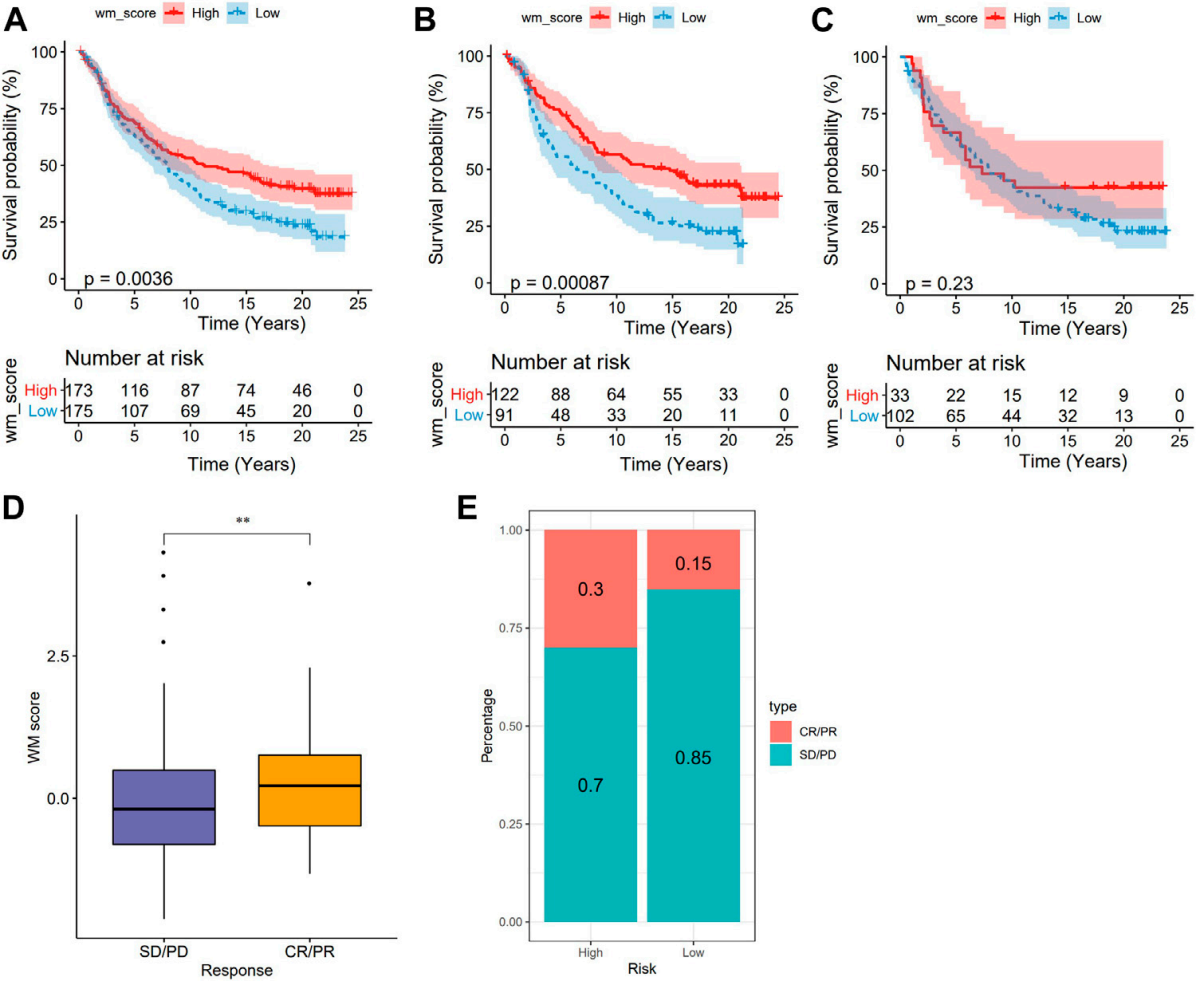
The WM score predicts immunotherapeutic benefit from anti-PD-1/L1 immunotherapy. **(A)**: Survival Analysis of high and low WM score groups in anti-PD-L1 cohort. **(B)**: Kaplan-Meier curves of two types of WM score in patients at stage I-II. **(C)**: Difference of OS between high and low WM score in patients with stage III-IV. **(D)**: Differences of WM scores between different anti-PD-L1 clinical response groups (SD: stable disease; PD: progressive disease; CR: complete response; PR: partial response). **(E)**: The fraction of patients with response to anti-PD-1 immunotherapy in low or high WM score groups. **p < 0.01.

In recent years, a number of clinical studies have reported that immunotherapy such as immune checkpoint inhibitors (ICIs) is effective in cancer treatment (Zhu et al., 2020). We also studied whether WM score can be applied to predict the response of LUAD patients to ICIs. In the anti-PD-L1 cohort, patients with a high WM score tended to develop a better prognosis than those with a low score (Figure 7A). According to the stratification of patients based on clinical stage, there was a significant difference in OS between patients with stage I-II and 2 MW scores, which was similar to that of Figure 7A (Figure 7B). However, for patients with stage III-IV, there was no significant difference in OS between high and low WM scores (Figure 7C). Therefore, WM score may be more suitable for early clinical prediction of LUAD. Moreover, the significant therapeutic effects and clinical response to PD-L1 treatment were confirmed in patients with high WM scores when compared to those with low WM scores (Figures 7D,E). These results suggested that WM score was related to LUAD patients’ response to immunotherapy and can be used to predict the prognosis of LUAD.

## Discussion

RNA modification induced by “writers” is the main contributor to post-transcriptional regulation of gene expression, and can occur in all RNA species, including messenger RNAs (mRNAs) and noncoding RNAs (ncRNAs) (Destefanis et al., 2021). Some of the known disorders of RNA modification and RNA modification “writers” have been found to be associated with various types of cancers, including breast cancer, bladder cancer, and leukemia (Jonkhout et al., 2017). The present research studied the genetic variation and expression changes of four kinds of RNA modification “writers” in LUAD. Two RNA modification patterns were determined based on 26 known RNA modification “writers”. Cluster_1 was significantly enriched to the pathways related to cell cycle, cell proliferation, and metabolism, and the abnormal activity of these pathways will lead to tumorigenesis (DeBerardinis et al., 2008; Kroemer and Pouyssegur, 2008; Eymin and Gazzeri, 2010; Cantor and Sabatini, 2012; Currie et al., 2013). This also supported the survival results that cluster_1 was worse than cluster_2 (cluster_2 was associated with heart disease, diabetes, and other diseases).

We have also developed a WM score to assess the RNA modification pattern of a single LUAD sample. The WM score showed a certain degree of independence and accuracy in predicting LUAD prognosis. LUAD patients with high WM scores tended to develop a poor prognosis and a higher proportion of plasma cells, CD8 T cells, activated memory CD4 T cells, resting NK cells, M0 macrophage, M1 macrophage, activated mast cells, neutrophils. Early studies showed that activated memory CD4 T cells and M0 macrophages are significantly infiltrated in high-risk LUAD (Mo et al., 2020). In addition, resting NK cells and activated plasma cells were also reported to have higher rates in high-risk non-small cell lung cancer (NSCLC) (Li et al., 2020). The densities of tumor-associated neutrophils in NSCLC were related to adverse prognostic factors (Carus et al., 2013). These studies also support the results of our analyses. For patients with low WM scores, the proportion of resting CD4 memory T cells, gamma delta T cells, activated NK cells, monocytes, resting mast cells and eosinophils were higher, and they also had higher stromal score and ESTIMATE score. These results validated that the WM score was related to TME.

Genetically, LUAD is a highly heterogeneous malignancy. Studies in the past few years have identified a large number of somatic mutations in LUAD (Testa et al., 2018). Mutations in TTN, TP53, and MUC16 are reported to be common in most types of cancer (Kim et al., 2013). Similarly, we found that these three genes were frequently mutated in LUAD patients with high WM scores, and that the TMB of high WM score samples were also higher at the same time. Evidence demonstrated that tumor genomic somatic mutations are associated with immune checkpoint inhibitor (ICI) treatment response (Samstein et al., 2019). We examined the responses of different WM score patients to anti-PD-L1 treatment and confirmed the significant therapeutic effects of PD-L1 treatment and clinical response of patients with high WM when compared to those with low WM scores. In addition, the responses of different patients with WM score to conventional drug therapy was explored, and the data revealed that drug sensitivity related to the WM score mainly involved the EGFR signaling pathway and that drug resistance related to the WM score mainly targeted the regulation of DNA replication, cell cycle, mitosis, and so on. Patients with a high WM score were more sensitive to four conventional treatments, namely erlotinib, XA V939, gefitinib, and KU-55933. These results indicated that RNA modification patterns could affect the therapeutic efficacy of ICI and cell line drug therapy.

In summary, our study developed a WM score based on differences in RNA modification patterns, and it can be used to evaluate RNA modification patterns, TME cell infiltration characteristics, clinical characteristics (AJCC stage, T stage, and N stage), genetic variation, and the response of patients with LUAD to ICI therapy. The current discoveries demonstrated the great potential of RNA modification patterns in predicting LUAD prognosis and in studying cancer cells in the future.

## Data Availability

The datasets presented in this study can be found in online repositories. The names of the repository/repositories and accession number(s) can be found in the article/Supplementary Material.
