# Corrigendum: The Identification of Two RNA Modification Patterns and Tumor Microenvironment Infiltration Characterization of Lung Adenocarcinoma

**DOI:** 10.3389/fgene.2022.879922

**Published:** 2022-03-11

**Authors:** Wan He, Gengpeng Lin, Chaohu Pan, Wenwen Li, Jing Shen, Yangli Liu, Hui Li, Dongfang Wu, Xuejia Lin

**Affiliations:** ^1^ Department of Oncology, Shenzhen People’s Hospital, Shenzhen, China; ^2^ Department of Pulmonary and Critical Care Medicine, The First Affiliated Hospital of Sun Yat-sen University, Institute of Pulmonary Diseases, Sun Yat-sen University, Guangzhou, China; ^3^ Zhuhai Institute of Translational Medicine, Zhuhai People’s Hospital (Zhuhai Hospital Affiliated With Jinan University), Jinan University, Zhuhai, China; ^4^ The Biomedical Translational Research Institute, Faculty of Medical Science, Jinan University, Guangzhou, China; ^5^ YuceBio Technology Co., Ltd., Shenzhen, China; ^6^ Department of Pathology, The First Affiliated Hospital of Sun Yat-sen University, Guangzhou, China

**Keywords:** RNA modification “writers”, lung adenocarcinoma, WM score, tumor microenvironment, immunotherapy

In the original article, we neglected to include the funder “Guangdong Finance Foundation for Industrial Technology Research and Development, 20160907 to Gengpeng Lin.” A correction has been made to the funding statement as given below:

“This study was supported by Shenzhen Science and Technology Innovation Commission (JCYJ20190807150403655), the Health, Population and Family Planning Commission of Shenzhen Municipality (SZXJ2018023), Young and Middle-aged Scientific Research Backbone Cultivation Project of Shenzhen People’s Hospital (SYKYPY201919) and Guangdong Finance Foundation for Industrial Technology Research and Development (20160907).”

The authors apologize for this error and state that this does not change the scientific conclusions of the article in any way. The original article has been updated.

