# Supplementary figures and images for: The Identification of Two RNA Modification Patterns and Tumor Microenvironment Infiltration Characterization of Lung Adenocarcinoma
Source: Front Genet. 2022 Jan 28;13:761681. doi: 10.3389/fgene.2022.761681 (PMC8831702; doi:10.3389/fgene.2022.761681)

Correlation with immune cell type

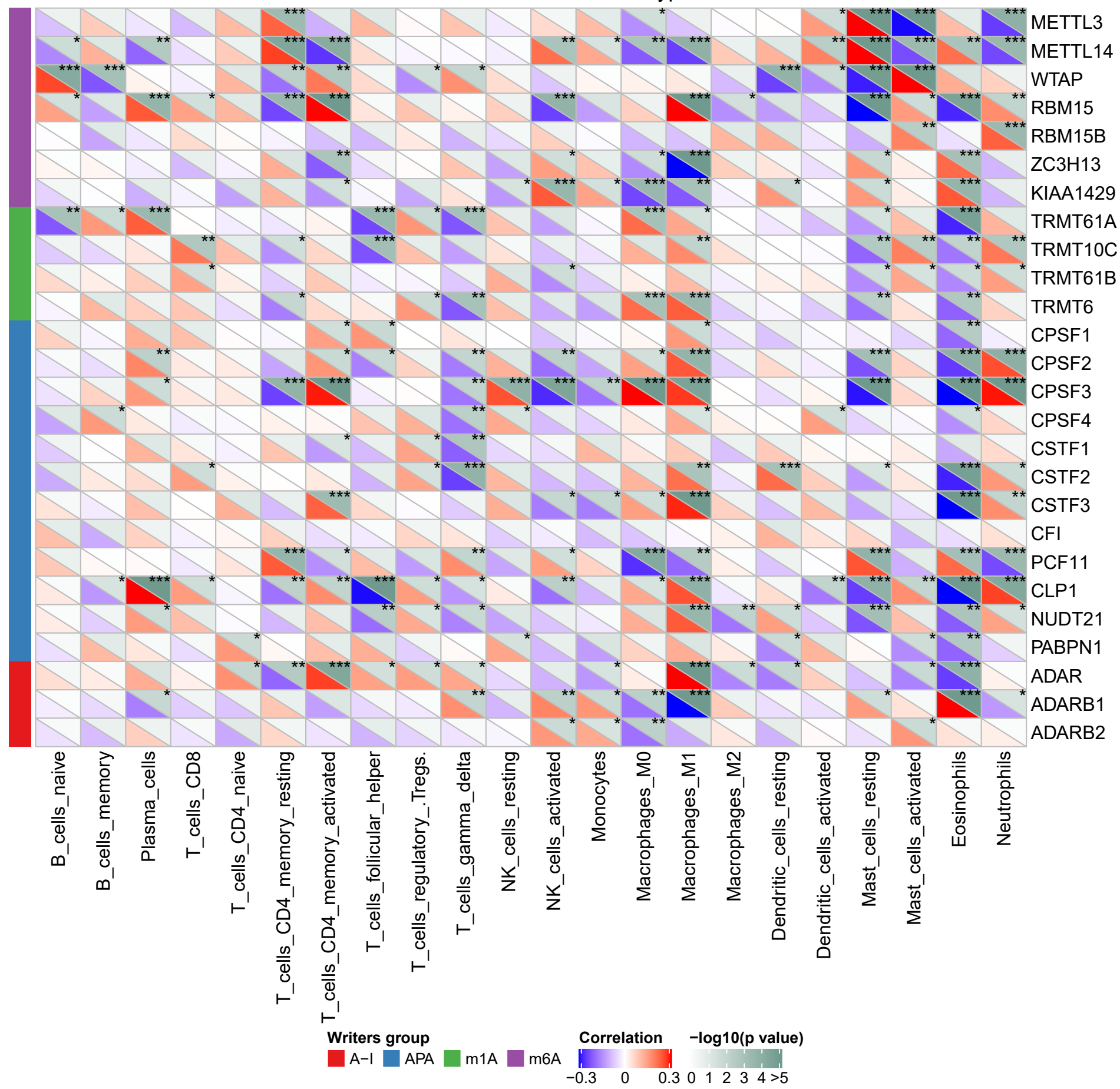

Supplement: Supplementary file 1 [file DataSheet2.PDF]

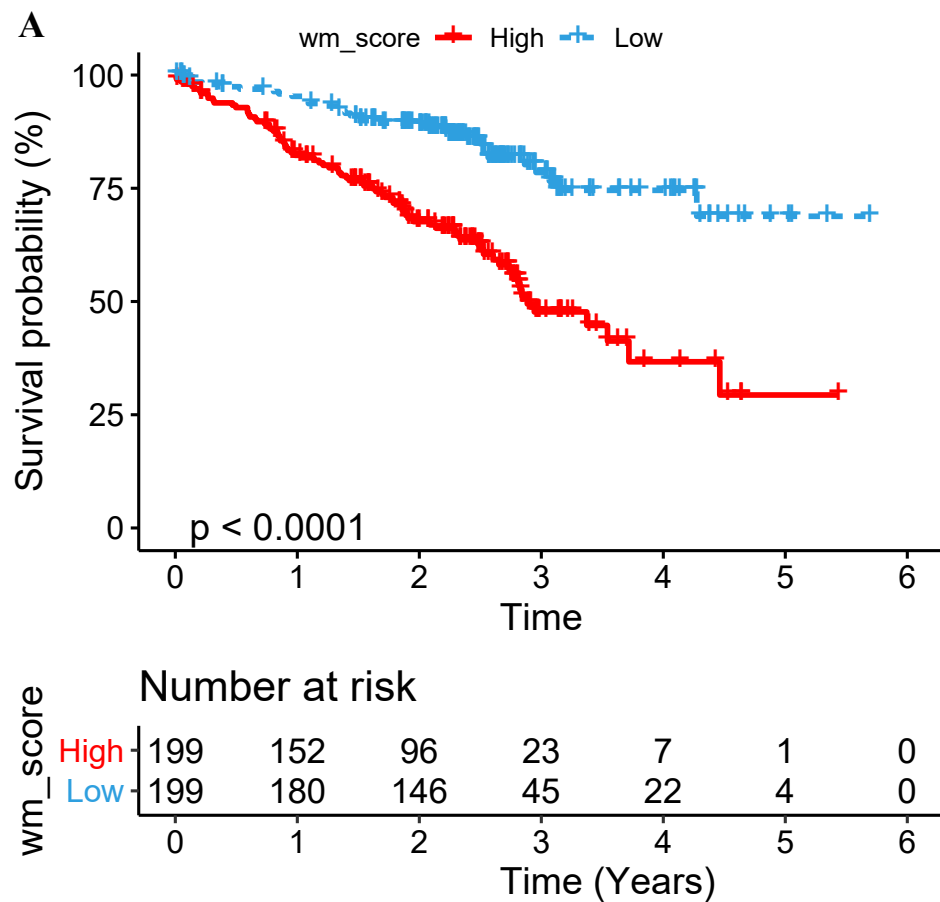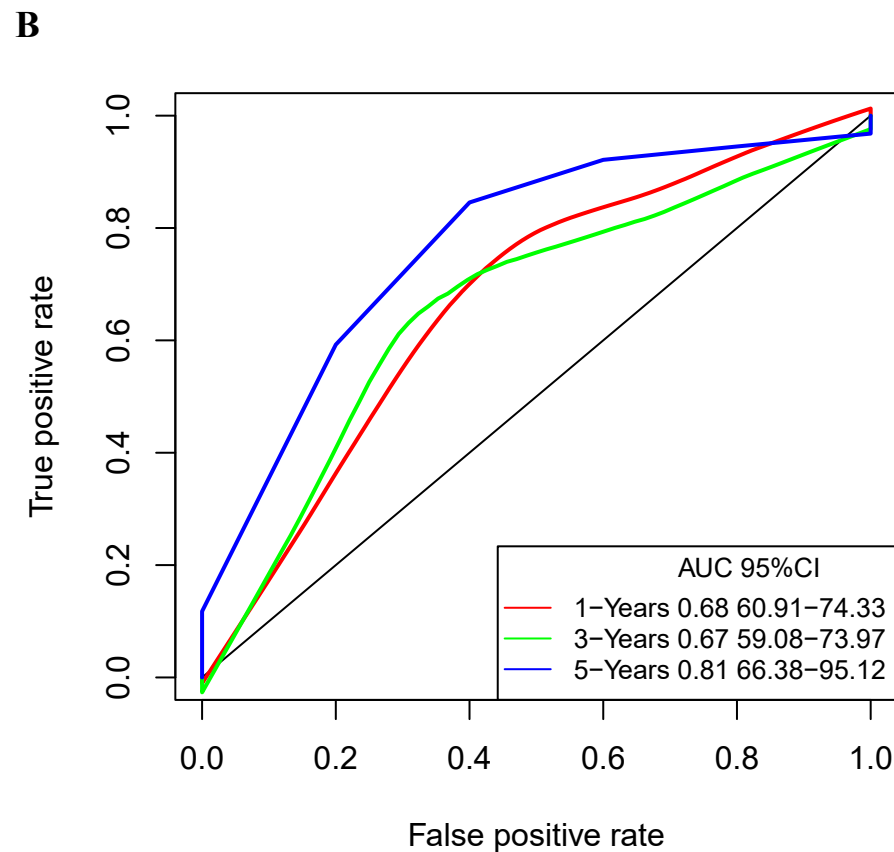

Supplement: Supplementary file 4 [file DataSheet3.PDF]

wilcox.tests p=1.1e-15

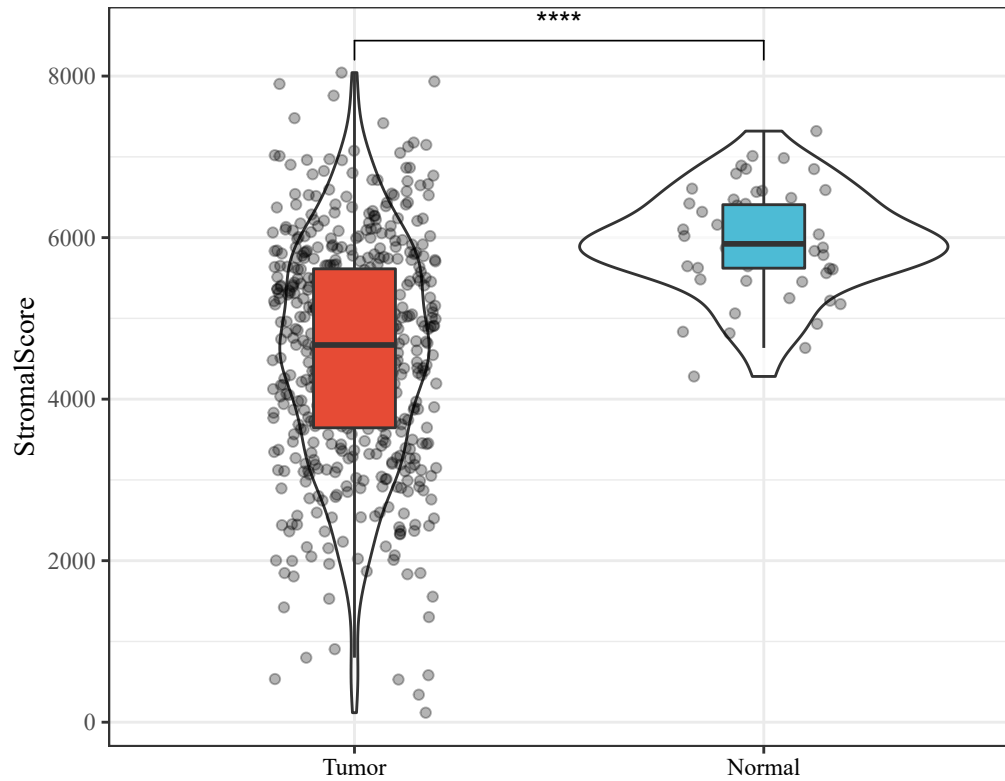

wilcox.tests p=5.4e-18

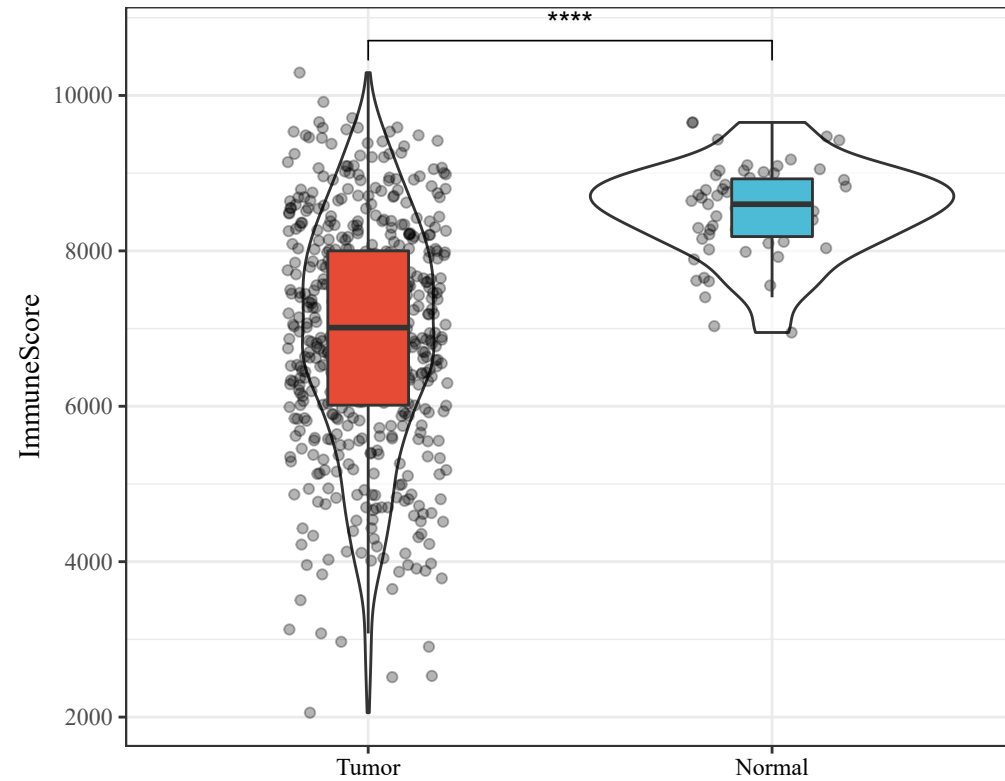

wilcox.tests p=3.9e-19

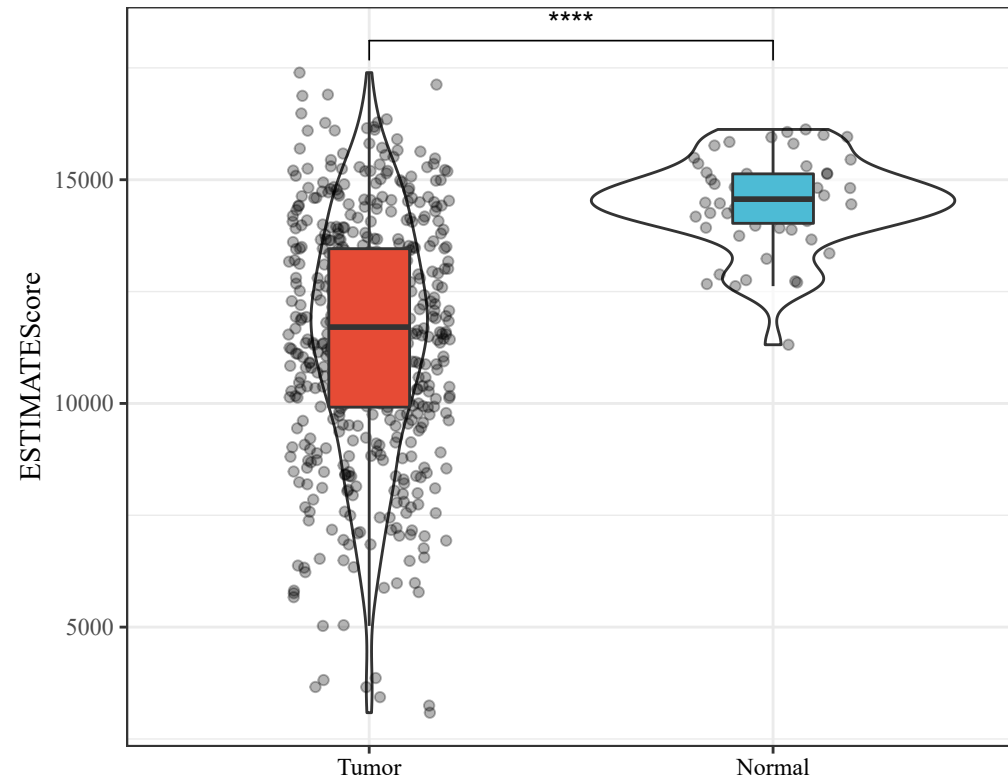

Group ■ Tumor ■ Normal

Supplement: Supplementary file 5 [file DataSheet1.PDF]
