# Supplementary material for: The Identification of Two RNA Modification Patterns and Tumor Microenvironment Infiltration Characterization of Lung Adenocarcinoma
Source: Front Genet. 2022 Jan 28;13:761681. doi: 10.3389/fgene.2022.761681 (PMC8831702; doi:10.3389/fgene.2022.761681)
Supplement: Supplementary file 2 [file DataSheet4.docx]

**Description of supplementary materials**

Figure S1 Differences in immune score, stromal score and ESTIMATE score between normal tissues and LUAD tissues in TCGA data set. **** P < 0.0001.

Figure S2 The correlation between RNA modification "writers" and TME cells. * P < 0.05; ** P < 0.01; *** P < 0.001.

Figure S3 The accuracy of WM score model was verified in GSE72094 cohort.

A: Kaplan-Meier survival analysis of two WM score subgroups. B: ROC curves for the prognostic performance of the WM score model in the GSE72094 cohort.

Table S1. 25 differentially expressed miRNAs between high- and low-WM scores.
