# Supplementary material for: The Identification of Two RNA Modification Patterns and Tumor Microenvironment Infiltration Characterization of Lung Adenocarcinoma
Source: Front Genet. 2022 Jan 28;13:761681. doi: 10.3389/fgene.2022.761681 (PMC8831702; doi:10.3389/fgene.2022.761681)
Supplement: Supplementary file 3 [file Table1.docx]

**Table S1. 25 differentially expressed miRNAs between high- and low-WM scores.**

| Seed+m8 | AveExpr | P.Value | adj.P.Val |
| --- | --- | --- | --- |
| miR-30-5p | -1.14146 | -11.834 | 1.45E-28 |
| miR-30-3p | -1.08587 | -11.1811 | 5.56E-26 |
| miR-99-5p/100-5p | -1.0128 | -12.2766 | 2.33E-30 |
| miR-105-5p | 1.726115 | 5.944971 | 5.29E-09 |
| miR-196-5p | 1.024541 | 3.486528 | 0.000534 |
| miR-210-3p | 1.509149 | 10.44757 | 3.50E-23 |
| miR-1-3p/206 | -1.43312 | -11.6011 | 1.24E-27 |
| miR-133a-3p.2/133b | -1.41238 | -13.0125 | 2.04E-33 |
| miR-9-5p | 1.878349 | 8.5741 | 1.35E-16 |
| miR-9-3p | 1.164989 | 7.488166 | 3.32E-13 |
| miR-184 | -1.15399 | -7.95602 | 1.26E-14 |
| miR-34b-5p/449c-5p | -1.14117 | -7.07513 | 5.25E-12 |
| miR-34-5p/449-5p | -1.59789 | -8.59207 | 1.18E-16 |
| miR-135-5p | -1.04287 | -7.08787 | 4.83E-12 |
| miR-196-5p | 1.486718 | 6.687672 | 6.26E-11 |
| miR-508-3p | -1.26892 | -6.41657 | 3.32E-10 |
| miR-509-3p | -1.00913 | -5.54788 | 4.76E-08 |
| miR-514a-3p | -1.23453 | -6.42833 | 3.09E-10 |
| miR-767-5p | 1.439085 | 5.816553 | 1.09E-08 |
| miR-29b-2-5p | -1.07374 | -12.1904 | 5.24E-30 |
| miR-30c-3p/6788-5p | -1.10347 | -10.2934 | 1.31E-22 |
| miR-34b-3p | -1.30259 | -8.32562 | 8.61E-16 |
| miR-34c-3p | -1.25475 | -7.02153 | 7.44E-12 |
| miR-3065-3p | -1.364 | -8.94475 | 7.88E-18 |
| miR-1247-3p | -1.0157 | -6.73891 | 4.54E-11 |
